# The patient costs of care for those with TB and HIV: a cross-sectional study from South Africa

**DOI:** 10.1093/heapol/czw183

**Published:** 2017-02-15

**Authors:** Don Mudzengi, Sedona Sweeney, Piotr Hippner, Tendesayi Kufa, Katherine Fielding, Alison D Grant, Gavin Churchyard, Anna Vassall

**Affiliations:** 1 The Aurum Institute, Johannesburg, 29 Queens Road, Parktown Johannesburg, Gauteng, 2193 South Africa; 2 Department of Global Health and Development, London School of Hygiene and Tropical Medicine, London WC1E 7HT, UK; 3 Department of Infectious Disease Epidemiology, London School of Hygiene and Tropical Medicine, Keppel Street, London WC1E 7HT, UK; 4 Department of Clinical Research, London School of Hygiene and Tropical Medicine, London WC1E 7HT, UK

**Keywords:** HIV, integration, patient costs, poverty, tuberculosis

## Abstract

**Background:**

This study describes the post-diagnosis care-seeking costs incurred by people living with TB and/or HIV and their households, in order to identify the potential benefits of integrated care.

**Methods:**

We conducted a cross-sectional study with 454 participants with TB or HIV or both in public primary health care clinics in Ekurhuleni North Sub-District, South Africa. We collected information on visits to health facilities, direct and indirect costs for participants and for their guardians and caregivers. We define ‘integration’ as receipt of both TB and HIV services at the same facility, on the same day. Costs were presented and compared across participants with TB/HIV, TB-only and HIV-only. Costs exceeding 10% of participant income were considered catastrophic.

**Results:**

Participants with both TB and HIV faced a greater economic burden (US$74/month) than those with TB-only (US$68/month) or HIV-only (US$40/month). On average, people with TB/HIV made 18.4 visits to health facilities, more than TB-only participants or HIV-only participants who made 16 and 5.1 visits, respectively. However, people with TB/HIV had fewer standalone TB (10.9) and HIV (2.2) visits than those with TB-only (14.5) or HIV-only (4.4). Although people with TB/HIV had access to ‘integrated’ services, their time loss was substantially higher than for other participants. Overall, 55% of participants encountered catastrophic costs. Access to official social protection schemes was minimal.

**Conclusions:**

People with TB/HIV in South Africa are at high risk of catastrophic costs. To some extent, integration of services reduces the number of standalone TB and HIV of visits to the health facility. It is however unlikely that catastrophic costs can be averted by service integration alone. Our results point to the need for timely social protection, particularly for HIV-positive people starting TB treatment.

Key Messages

- People with both TB and HIV in South Africa are at high risk of catastrophic costs.
- Integration of services has to some extent reduced the number standalone TB and HIV visits to the health facility. It is, however, unlikely that catastrophic cost can be averted by service integration alone.
- Social and income protection policies are likely to be required to protect TB/HIV patients if global targets on catastrophic cost reduction are to be met.

## Introduction

The launch of the sustainable development goals and Universal Health Coverage reflect an increased global focus on the interaction between health outcomes and poverty. Health sector policymakers are becoming increasingly interested in interventions and service delivery models that may best prevent impoverishment. While there has been much investigation into the impact of service integration on provider costs, much less attention has been focussed on the potential economic and poverty reduction benefits to service users, particularly vulnerable groups (World Bank 2009; World Health Organization *et al.* 2009; Atun *et al.* 2010).

Household and patient-incurred costs associated with health shocks have long been recognized as key contributors to impoverishment (Heltberg and Lund 2009; Alam and Mahal 2014; Wagstaff and Lindelow 2014). In the case of TB, patient-incurred costs are a major barrier to access to health services in low-income countries (Ensor and Cooper 2004; O’Donnell *et al.* 2007), and have been associated with negative TB treatment outcomes (Wingfield *et al.* 2014). Even where TB services are offered free of charge, the high costs of access such as transportation and opportunity cost of time spent accessing care may provide obstacles for vulnerable groups, while worsening or creating poverty in those that proceed to seek care (Xu *et al.* 2007). When faced with high costs of accessing TB care and a reduced ability to earn income due to illness, some TB patients resort to selling off their assets and taking interest-bearing loans (Lönnroth *et al.* 2014). This can result in a long-term poverty impact for both patients and their households (Xu *et al.* 2003; Gottret and Schieber 2006; WHO 2010; Lönnroth *et al.* 2014).

For people accessing care for both TB and HIV, health service integration has the potential to reduce this economic burden. Integration may benefit patients by enabling health improvements and cost reductions through less fragmented services, improved continuity of care and better retention in care (Sweeney *et al.* 2012). Integration may also facilitate cost reductions through fewer visits to facilities and reduced delays in accessing treatment (Legido-Quigley *et al.* 2013).

In 2012, TB was the primary cause of death for 25% of all HIV-associated deaths in South Africa, and 61% of all people with TB were HIV-positive (WHO 2015). The country has developed guidelines for the integration of TB and HIV services with preference for a ‘one-stop shop’, where services are provided under one roof (National Department of Health 2011). TB/HIV integration is expected to ‘ensure comprehensive management of the patient, reduce morbidity and mortality and improve treatment outcomes’ (Chehab *et al.* 2013; National Department of Health 2014). Integration however remains poorly implemented in South Africa (Churchyard *et al.* 2014). Although services are commonly provided ‘under one roof’, they may often not be provided by a single provider, nor will patients be correctly referred between providers. As a result, the evidence base on the impact of TB/HIV integration on patient-relevant outcomes is small and inconsistent (Kaplan *et al.* 2014; Jacobson *et al.* 2015; Ledibane *et al.* 2015).

To date, TB patient costing studies in South Africa (Chimbindi *et al.* 2015; Foster *et al.* 2015) have not comprehensively assessed the economic impact of illness on people with both TB and HIV. The purpose of this paper is to comprehensively describe the post-diagnosis care-seeking behaviour, patient costs incurred and coping strategies adopted by people living with TB and/or HIV and their households, in order to identify the potential benefits of integrated care. To present this, we collected data on the costs incurred by participants in the period immediately following receipt of a TB and/or HIV diagnosis and including the first 3–5 months of care, as this is the period when previous studies have shown patients to incur the highest costs (Foster *et al.* 2015). We present this evidence in order to support policy makers as they assess the potential benefits from the improved implementation of TB/HIV integration.

## Methods

### Study setting

The study was conducted in Ekurhuleni North; a sub-district in Gauteng province, South Africa. Ekurhuleni had approximately 3.2 million inhabitants in 2013 (City of Ekurhuleni 2013) and a population density of approximately 1609 people per square kilometre (Statistics South Africa 2015). Ekurhuleni has high unemployment rates of 28.8% in the general population and 36.9% among persons between the ages of 15 and 35 (City of Ekurhuleni 2013, 2014). In 2013, 8% of the people living in Ekurhuleni reported that they did not have any source of income and 27.9% were considered to be living below a nationally defined minimum living standard (City of Ekurhuleni 2013). The South Africa District Health Barometer of 2013 estimated a TB case notification rate of 336 per 100 000 for Ekurhuleni (Massyn *et al.* 2014). According to a national HIV prevalence, incidence and behaviour survey, the HIV prevalence for Ekurhuleni was 14.3% (10.3–19.5%) in 2012 (Simbayi *et al.* 2014).

### Study design and baseline data collection

This was a cross-sectional study nested within a cluster randomized trial—the MERGE trial. The MERGE trial evaluated the effect of implementing an intervention to optimize/improve TB/HIV integration on morbidity, mortality and retention in care at public primary health care (PHC) clinics (Kufa *et al.* 2014). A total of 18 PHC clinics, the study clinics, were randomly allocated to the intervention or control arm. To be eligible for inclusion in the trial, the clinics had to meet the following criteria: no conflicting research study in progress at the clinic, clinic has at least 40 TB cases per year and the clinic has available TB data.

Participation in the MERGE trial was not a requirement for inclusion in the patient costs study. Instead MERGE trial participants had an equal chance of also being enrolled in the patient costs study if eligible. Cost data were collected using structured questionnaires at the 18 study clinics between April and October 2013. Participants were selected consecutively and enrolled if they met any one of the following criteria: (1) received a TB diagnosis 3–5 months prior to interview and had a positive HIV test at any time (‘TB/HIV’); (2) received a TB diagnosis 3–5 months prior to interview and was HIV negative at time of enrolment (‘TB-only’); and (3) tested HIV positive for the first time 3–5 months prior to interview and was not on treatment for TB at the time of enrolment (‘HIV-only’). The time period was informed by previous research that showed that participant recall becomes diminished at around 4 months onwards (Mauch *et al.* 2011). All participants reported a known positive or negative HIV status. Unlike TB, HIV positive reporting was not confirmed with clinic records. Participant numbers were capped at 50 per site, although only 3 of 18 sites reached this cap due to low participant numbers at the facilities.

### Questionnaires

Questionnaires were adapted from the Tool to Estimate Patients’ Costs that was developed by the Tuberculosis Coalition for Technical Assistance and the United States Agency for International Development (USAID and TBCTA 2008). Separate questionnaires were developed for people being treated for TB (regardless of HIV status) and for HIV-positive people not being treated for TB to accommodate different pathways of care. Both questionnaires captured similar level of detail on the different events in the pathway of care. Questionnaires focused on the period in the first 3–5 months after participants were told they had TB (‘post-diagnosis’) to understand the costs of accessing integrated services.

Demographic characteristics such as gender, age, ethnicity and nationality, levels of education, marital status, employment at the time of receipt of diagnosis and the impact of illness on normal productive patterns were collected. Questionnaires also included detailed questions on the number of visits made to a range of providers, including the participant’s local PHC clinic (our study clinic), other public facilities, general practitioners, hospitals, traditional healers and pharmacies. A distinction was made between integrated visits and stand-alone visits for TB and/or HIV services at the study clinic. We define ‘integration’ as physical and temporal integration, or receipt of both TB and HIV services at the same facility, on the same day (Mayhew *et al.* 2016).

It was not feasible to measure costs for every visit made by participants; questions therefore elicited estimates of direct costs, time spent and income loss for the most recent visit to each provider, and the number of visits made to each provider type during the treatment period. The questionnaires also captured information about strategies adopted by participants to cope with costs of illness. Coping strategies enquired of include: taking interest-bearing loans from lenders, borrowing money from friends or relatives, selling personal goods and receipt of grants or charitable donations.

### Data analysis

The data were captured in a secure electronic database and exported into Stata 14 and Microsoft Excel for analysis (Microsoft 2014; Stata Corp 2015). An ‘available case analysis’ assumed that unavailable data values were missing at random. All costs were converted to an average monthly cost to facilitate comparison across participants who had received diagnosis between 3 and 5 months prior to interview.

Direct costs were defined as medical and non-medical expenses paid out-of-pocket (OOP). Medical expenses included consultation fees and any OOP payment for medicines and diagnostics paid at any provider. Direct non-medical expenses included the travel costs of participants and guardians if any, food costs incurred while in hospital, money spent buying any special foods or dietary supplements due to illness, and any interest incurred on loans taken out to meet the costs of OOP payments. Direct medical and non-medical costs were determined as the product of the reported expense for the most recent visit to each provider type and the number of visits made to that provider during the post-diagnosis period; these were then divided by the number of months in the post-diagnosis period.

We use reported income loss as our primary measure of indirect costs for participants. To facilitate comparison with other patient cost studies, we also report separately on time the participants spent seeking care or unable to work. We estimated the mean time spent per month using the total time reported for the most recent visit to each provider, multiplied by the total monthly visits to each provider. Indirect costs for guardians and carers were defined as the opportunity cost of time spent away from their daily productive routine, including travel to health facilities, consultation time, and covering household chores usually done by the participant. As guardians and carers were not interviewed directly about their income loss, the opportunity cost of this time for guardians and carers was estimated using median income of elementary occupations in South Africa, R 1517 per month (Statistics South Africa 2010) multiplied by their estimated time loss. Loan costs were calculated as the difference between the borrowed amount and the amount paid back.

We also estimated catastrophic costs incurred due to TB and/or HIV. The principle of catastrophic costs is rooted in identifying when patients and their households involuntarily reduce expenditure on basic household needs such as food, clothing and education in order to pay for health care (Ranson 2002). According the World Health Organization (WHO) approach, costs are defined as catastrophic when total costs incurred (direct and indirect combined) exceed a given threshold of household income (World Health Organization 2015). In the absence of reliable data on household income, we adopted a threshold of 10% of individual participant income (Barter *et al.* 2012). This threshold has been a widely used benchmark for catastrophic costs in many patient costing studies (Xu *et al.* 2003; Russell 2004; Tanimura *et al.* 2014; Wingfield *et al.* 2014; Foster *et al.* 2015) due to the challenges of measuring household rather than individual income. An alternative 20% threshold of household income is also being increasingly used in the case of TB, due to an observed association between this level of cost and negative health outcomes in Peru (Wingfield *et al.* 2014). We varied the catastrophic cost threshold in our analysis from 5 to 25% to understand the impact of this arbitrary threshold (Russell 2004; Ukwaja *et al.* 2013). To avoid mathematical errors associated with division by zero, an arbitrary value of USD1 was assigned to income for those participants who reported zero income or where income was a missing value (Foster *et al.* 2015).

We adopted a descriptive cost analysis due to the small sample size of some of the comparison groups. Prior to analysis, all costs were converted from the South African rand (ZAR) to the United States dollar (USD) using the average rate during the period of data collection in 2013; ZAR 9.62 =  US$1 (OANDA 2016). Despite skewness and non-normality of cost data, arithmetic means were used in all calculations as was done in the previous studies (Wingfield *et al.* 2014) and in line with the principles of economic evaluation (Bill and Melinda Gates Foundation et al. 2014). Standard deviations were used as measures of dispersion for cost data and inter-quartile ranges for continuous descriptive data.

### Ethical considerations

Ethical approval was obtained from The London School of Hygiene & Tropical Medicine and the University of the Witwatersrand. The study was also registered in the clinical trials register for South Africa (registration number DOH-27-10113846) and additional permission to conduct the study was sought from the Ekurhuleni health department.

## Results

We invited 475 participants meeting the inclusion criteria to participate in the study and 463 consented to participate. The most common reason for non-inclusion was receipt of diagnosis outside of the window of 3–5 months prior to interview. Of the 463 enrolled, 454 participants from 18 PHC clinics were included in the analysis, with 9 participants excluded because data on their gender were missing at analysis stage. The majority of the participants included in the analysis had received a diagnosis of HIV-only (*n*  =  298; 66% of sample). Forty TB-only participants and 116 TB/HIV participants were recruited. Of the TB/HIV participants, 20 received both TB and HIV diagnoses on the same day and an additional 46 received both diagnoses within 2 months of each other.

### Descriptive characteristics

Characteristics of the study population are presented in Table 1. The majority of participants was unmarried (58%). Most participants were female (64%) and educated above grade 8 (84%). Participants born in South Africa and those of African origin made up 83 and 97% of the study population, respectively. Unemployment was very high across all participant groups; 45% of enrolled participants were unemployed at the time of receiving their diagnosis, as compared to a national unemployment rate of 25% (Statistics South Africa 2015). Median monthly income was $128 at the time of diagnosis with TB and/or HIV. Of those who were employed at the time of diagnosis, 6% had a monthly income below the national poverty line of $52 per month (Statistics South Africa 2014). The highest income at the time of diagnosis was reported by the TB/HIV group (median $150 per month), while the TB-only group had the lowest average income ($88 per month).

**Table 1.** Demographic characteristics at time of interview, by participant group

		TB/HIV		TB-only		HIV-only	
		(*n* = 116)		(*n* = 40)		(*n* = 298)	
**Female, *n* (%)**		63	22%	16	6%	210	73%
**Age, *n* (%)**	18–24	10	9%	5	13%	22	7%
	25–34	43	37%	21	53%	131	44%
	35–44	48	41%	6	15%	96	32%
	≥45	13	11%	8	20%	43	14%
**South African, *n* (%)**		100	86%	32	80%	244	82%
**Black/African, *n* (%)**		111	96%	38	95%	291	98%
**Grade 8 and above, *n* (%)**		95	82%	34	85%	251	84%
**Unmarried, *n* (%)**		69	59%	24	60%	169	57%
**Employed at diagnosis, *n* (%)**		60	52%	20	50%	168	56%
**Had informal carers in post-diagnosis period, *n* (%)**		56	48%	22	55%	111	37%
**Missed work in post-diagnosis period, *n* (%)**		36	31%	11	28%	37	12%
**Median CD4 count at last test (IQR)**		125	275			244	216
**Median monthly income at diagnosis (2012 USD) (IQR)**		$150	381	$88	342	$135	312
**Median days from diagnosis to interview (IQR)**		115	28	119	32	115	33

A large proportion of participants had informal carers; 55% of those with TB-only, 37% of those with HIV-only and 48% of those with TB/HIV. The impact of illness and care-seeking had variable effects on participants’ and household members’ income-earning activities. Across all participant groups, 19% of participants missed work due to illness and 21% of participants were unable to complete their normal household duties in the post-diagnosis period. People with TB were more likely to miss work with the highest proportion being 31% among TB/HIV participants.

### Health service use

All study facilities offered integrated care for both TB and HIV as defined in the methods section. Actual practice at study facilities varied considerably; in some facilities visits were integrated at the provider level where both services were delivered by the same provider or the consultation level where both services were delivered within the same consultation, though the latter was rare. Table 2 shows the overall mean number of clinic visits and by visit type, for each participant group. TB/HIV participants on average made five ‘integrated’ visits in the post-diagnosis period. TB-only participants also received integrated visits when testing for HIV and collecting test results—on average this was 0.8 visits per person across study facilities.

**Table 2.** Visits to any health care provider in the post-diagnosis period, by participant group

Patient group	TB/HIV			TB-only			HIV-only		
	(*n* = 116)			(*n* = 40)			(*n* = 298)		
Visit type	TB/HIV visits	TB visits	HIV visits	TB/HIV visits	TB visits	HIV visits	TB/HIV visits	TB visits	HIV visits
**Study clinic visits, mean (SD)**	5.0 (4.6)	10.9 (14.2)	2.2 (4.6)	0.8 (.6)	14.5 (14.6)	0.1 (.2)	0	0	4.4 (2.0)
**Visits to**^a^**other providers, mean (SD)**		0.3 (0.7)	0.2 (0.7)		0.6 (2.6)	0.1 (0.2)		0	0.2 (0.7)
**Subtotal, all providers, mean**	5.0	11.2	2.2	0.8	15.1	0.1	0	0	4.6
**Total visits, all visit types, all providers**	18.4			16.0			5.1		

Note: SD, standard deviation.

a Other public clinic, pharmacy, general practitioner, hospital-outpatient, hospital-inpatient and traditional healers.

In the post-diagnosis period, all participants made relatively few visits to providers outside the public health system. The average total number of visits to other facilities and providers ranged from 0.6 in the TB-only group to 0.2 in the HIV-only group. The total number of participants accessing other types of health provider, and mean number of visits by those participants, is presented in Supplementary Table S2. The largest proportion of participants accessing care from providers outside the public health system was among the TB/HIV participant group, at 23%. Fifteen percent of TB-only participants and 14% of HIV-only participants reported use of providers outside the public health system respectively. Thirteen TB/HIV participants were hospitalized, as compared to two HIV-only participants and zero TB-only participants.

All people with TB visited the study health facilities at least four times per month in the post-diagnosis period. HIV-only participants made the fewest visits to study facilities over the study period (mean 1 visit per month).

### Patient costs

Table 3 presents patient-incurred costs in the post-diagnosis period. The highest total costs in the post-diagnosis period were reported by TB/HIV and TB-only participants; $74.07 and $68.33 per month, respectively. Costs for the HIV-only group ($40.41 per month) were substantially lower. Indirect costs contributed the majority of the total costs, at 71% of total cost for TB/HIV participants, 86% of total cost for TB-only participants and 55% of total cost for HIV-only participants.

**Table 3.** Monthly direct and indirect costs (USD 2013), by participant group

	TB/HIV		TB-only		HIV-only	
	(*n* = 116)		(*n* = 40)		(*n* = 298)	
	Mean	(SD)	Mean	(SD)	mean	(SD)
**Direct costs**						
** **Patient medical						
** **Study clinic	0.00	0.00	0.00	0.00	0.00	0.00
** **Any other facility	1.71	10.23	0.07	0.42	0.87	4.12
** **Patient travel						
** **Study clinic	4.12	8.91	1.69	3.31	1.25	3.07
** **Any other facility	0.63	2.89	0.05	0.20	0.24	1.37
** **Guardian travel						
** **Study clinic	0.43	2.37	0.00	0.00	0.27	2.78
** **Any other facility	0.51	3.11	0.00	0.00	0.17	1.52
** **Food						
** **Hospital	0.26	1.31	0.00	0.00	0.04	0.47
** **Special foods	13.14	17.33	8.06	11.05	9.76	14.91
** **Loan interest	0.93	9.78	0.00	0.00	5.68	89.11
**Total direct costs**	21.72 (29%^a^)		9.86 (14%^a^)		18.28 (45%^a^)	
**Indirect costs**						
** **Patient income loss						
** **Job loss income loss	15.40	126.17	17.78	76.69	2.99	24.30
** **Care-seeking income loss	30.45	105.56	34.60	98.99	13.81	59.03
** **Opportunity costs of time						
** **Guardian						
** **Study clinic	1.13	2.37	0.23	0.00	3.92	2.78
** **Any other facility	0.94	6.16	0.04	0.24	0.22	1.24
** **Carer	4.42	11.35	5.81	13.52	1.19	5.77
Total indirect costs	52.34 (71%^a^)		58.47 (86%^a^)		22.13 (55%^a^)	
Grand total	74.07		68.33		40.41	

Note: SD, standard deviation.

a Percentage of the overall total.

Direct OOP costs incurred by participants ranged from $9.86 per month for TB-only participants to $21.72 per month for TB/HIV participants (Table 3). Detailed costs incurred at all facility types are listed in Supplementary Table S2. Direct costs were largely driven by costs of special food purchased as nutritional supplements for the illnesses in question. Monthly costs of special foods ranged from $8.06 to $13.40 per month, representing 53% of direct costs for HIV-only participants, 60% of direct costs for TB/HIV participants and 82% of direct costs for TB-only participants. Expenditure on special foods alone represented an average of 30, 13 and 27% of total income for HIV-only, TB-only and TB/HIV participants, respectively. None of the interviewed participants incurred direct medical costs at the study clinic, or at any other PHC clinic. Participants who sought care from health facilities outside the public health system, particularly those with TB/HIV, incurred some direct medical costs; an average of $1.71 per month was observed for TB/HIV participants. The highest direct medical costs from providers outside the public health system were incurred by participants accessing care from traditional healers, however this was driven by one participant reporting a very high cost of $415.

Indirect costs were high for all participant groups, particularly those participants being treated for TB. Job loss and other income losses were major drivers for indirect costs; accounting for 62% of cost in the participants with TB/HIV and 77% of cost for TB-only participants. Participants with TB (both TB-only and TB/HIV) lost an average of $32.53/month in income due to time spent seeking care. HIV-only participants lost substantially less income due to seeking care on average than other participant groups, at an average of $2.99/month.

About 4% of participants with TB and 3% of those with HIV lost their job entirely due to illness. Among those who lost their jobs due to illness, the mean and median income losses were $321.62 and $207.90, respectively. The average income loss due to job loss across all participants was $17.78/month for TB-only participants, $15.40/month for TB/HIV participants and $2.99/month for HIV-only participants. The monetary value of time lost by guardians was particularly high for HIV-only participants. Table 3 shows the monthly guardian opportunity costs of time varying from $2.07 in the TB/HIV group to $4.14 in the HIV-only group. In contrast, the cost of informal caregiving was particularly high for participants with TB (regardless of HIV status); this cost averaged $5.81 per month for TB-only participants and $4.42 per month for TB/HIV participants.

### Patient time loss

The time that participants lost while travelling to health facilities and accessing (and waiting for) care in the post-diagnosis period is presented in Table 4. TB/HIV participants lost the most time, averaging 91 h per participant over the post-diagnosis period. This was more than the combined time loss of TB-only and HIV-only participants (33.8 and 23.4 h, respectively). The time lost by TB/HIV participants was driven by long hospitalizations for 11 out of 116 (9.4%) participants who were hospitalized for an average of 17.7 nights over the post-diagnosis period. The average time loss for TB/HIV participants not hospitalized was 50 h over the post-diagnosis period. Travel time, particularly for visits to the study clinic, was also substantial. TB/HIV participants lost an average of 20 h travelling, while TB-only and HIV-only participants lost an average of 15 and 6 h travelling, respectively.

**Table 4.** Total time loss in post-diagnosis period (hours), by participant group

		TB/HIV		TB-only			HIV-only	
		(*n* = 116)		(*n* = 40)			(*n* = 298)	
		mean	(SD)	Mean	(SD)		mean	(SD)
Study clinic	Consulting	28.2	27.7	17.5	17.3		13.9	11.7
	Travel	20.7	20.7	15.4	17.3		5.6	6.4
	Subtotal	48.9 (54%)		32.9 (97%)			19.6 (83%)	
Other clinic	Consulting	0	0.2	0.3	1.0		0.1	0.5
	Travel	0.1	0.5	0.5	1.4		0.2	1.3
	Subtotal	0.1 (0%)		0.8 (2%)			0.2 (1%)	
Pharmacy	Consulting	0	0.2	0	0.1		0	0.4
	Travel	0	0.3	0	0.1		0.1	0.4
	Subtotal	0.1 (0%)		0 (0%)			0.1 (0%)	
General practitioner	Consulting	0.1	0.7	0	0.3		0.3	1.8
	Travel	0.2	1.2	0	0		0.2	0.8
	Subtotal	0.3 (0%)		0 (0%)			0.4 (2%)	
Hospital-inpatient	Consulting	40.3	146.8	0	0		2.9	25.5
	Travel	0.3	1.0	0	0		0.1	0.7
	Subtotal	40.6 (45%)		0 (0%)			3.0 (13%)	
Hospital-outpatient	Consulting	0.2	1.0	0	0		0	0
	Travel	0.5	3.2	0	0		0	0
	Subtotal	0.7 (1%)		0 (0%)			0 (0%)	
Traditional healer	Consulting	0	0.3	0	0		0	0.2
	Travel	0.1	0.8	0	0		0.1	0.9
	Subtotal	0.1 (0%)		0 (0%)			0.2 (1%)	
Grand total		90.8		33.8		23.4	

Note: SD, standard deviation.

### Catastrophic costs

**Figure 1.**
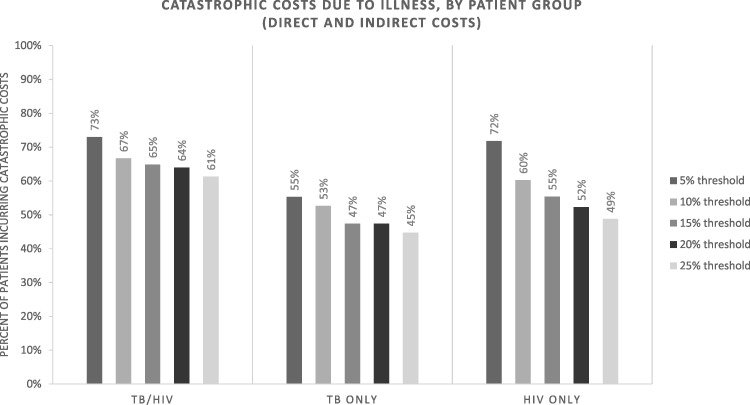
Catastrophic costs due to illness, by participant group

Figure 1 illustrates the percentages of participants facing catastrophic cost, varying thresholds from 5 to 25%. All participants had high rates of catastrophic expenditures, across thresholds. The results show that >60% of all participants face catastrophic costs at the 10% threshold. TB/HIV participants show the highest proportions facing catastrophic costs, with 73% of participants encountering catastrophic costs at the 5% threshold and 61% at the 25% threshold; >70% of HIV-only participants experienced catastrophic at 5% threshold, however this proportion dropped at higher thresholds. Considering only direct costs reduced the proportion of participants encountering catastrophic costs to 68–50% of TB/HIV participants, 46–31% of TB-only participants and 54–33% of HIV-only participants depending on threshold (Supplementary Figure S1).

### Coping strategies

Table 5 shows the range of strategies adopted by participants and their households to cope with income loss and/or direct out of pocket payments incurred due to TB and/or HIV (Table 5). Fifteen percent of HIV-only participants, 6% of TB/HIV participants and 8% of TB-only participants adopted at least one coping strategy. The most common coping strategy was loan-taking, which was done by 11% of HIV-only participants, 8% of TB-only participants and 3% of TB/HIV participants. Interest charged on loans to the TB/HIV and HIV-only group were relatively high, at 27 and 22% of the initial value, respectively. In contrast, TB-only participants were able to source loans at zero interest from friends or family. Government grants and charitable donations were rarely accessed across all participant groups. Similarly, asset sales were not used by the majority of participants as a means to cope with TB and/or HIV-related costs.

**Table 5.** Coping strategies, by participant group

	TB/HIV	TB-only	HIV-only
	(*n* = 116)	(*n* = 40)	(*n* = 298)
Grants and donations			
Patients receiving government grants n (%)	1 (1%)	0	5 (2%)
Patients receiving charitable donations n (%)	0 (%)	0	4 (1%)
Asset sale			
Patients selling assets n (%)	3 (3%)	0	7 (2%)
Mean value of assets sold (USD)	$11.54	–	$9.15
Loans			
Patients taking loans n (%)	4 (3%)	3 (8%)	34 (11%)
Mean interest on loans (% of initial withdrawal)	27%	0%	22%
Total adopting any coping strategy	7 (6%)	3 (8%)	46 (15%)
Total adopting multiple strategies	1 (1%)	0	4 (1%)

## Discussion

All participants interviewed in this study encountered high costs associated with HIV and/or TB. Over 45% of all participants experienced catastrophic costs even at thresholds as high as 25% of individual income. People with both TB and HIV on average face higher levels of post-diagnosis catastrophic costs than those with TB-only or HIV-only, especially at higher thresholds.

In principle, integration has the potential to reduce the overall number of visits. We found many participants were receiving integrated care, defined as receiving multiple services at the same facility on the same day. TB/HIV participants received an average of five ‘integrated’ TB/HIV visits in the post-diagnosis period, where both TB and HIV services were delivered on the same day. As a result, participants received fewer TB-only visits than the TB-only group, and fewer HIV-only visits than the HIV-only group. However, the total time loss for TB/HIV participants was still considerably higher than time loss for other participants. Similarly, travel costs for people with TB/HIV were substantially higher than all other participants. Given the high costs faced by those with TB/HIV, further gains may be achieved by ensuring that ‘integrated’ visits are delivered by the same provider or within the same room, reducing waiting periods between multiple visits in a day.

The reduced visits observed for people with both TB and HIV may be extended by further integration, where services are provided within the same visit, minimizing the need for separate appointments. However, given the existing level of integration in terms of numbers of joint TB/HIV visits, it is unlikely that catastrophic cost can be averted by integration alone, and our results point to the need for timely social protection schemes such the government temporary disability grant, particularly for HIV-positive people starting TB treatment.

To some degree, patients are able to cope with the costs of care, for example, through taking loans with little or no interest from family and friends. However, where costs are particularly high or where patients lack social capital, coping strategies may place patients at risk of worsened long-term economic burden. For example, access to loans in some instances can show a level of credit worthiness; particularly where loans are taken from family or friends with no interest they have been regarded in the literature as an indicator of social capital and a possible way for households to reduce the economic burden of illness (Chuma *et al.* 2007). However, where loans are taken out with high interest rates or where productive assets are sold, households face the risk of long-term economic hardship (Madan *et al.* 2015). The extent of loan-taking at high interest in order to meet the costs of health care suggests that people with HIV may be at high risk of long-term economic hardship. People with HIV were also more likely to sell assets in order to pay for care; this may also translate to diminished financial status because assets may have been sold for less than their replacement values.

In addition to loans and asset sale, some people received grants and donations to deal with costs of illness. Currently, the South African government offers a temporary social relief of distress grant for patients who at the discretion of a doctor are deemed unfit to undertake remunerative work (Department of Social Development 2006). However, access to these were consistently low, with 1% of participants overall accessing government grants. People with TB in particular had little access to the temporary disability grant, even when they were encountering catastrophic costs. This may be due to difficulty accessing the required certifications of disability within a rapid time frame. Access to charitable donations was similarly low, with only 4 of the 454 participants interviewed accessing a donation. This notable absence of donations and grants for all participants, and TB participants in particular, shows a policy implementation gap for the most vulnerable TB patients. Further research on the reasons for this implementation gap is needed, and the South African government should thus consider alternative social protection mechanisms, such as unconditional immediate cash transfers to TB patients to close this gap (Boccia *et al.* 2011).

Participants with HIV (both HIV-only and TB/HIV) encountered relatively high costs due to accompaniment by guardians to the study facility. South African HIV treatment policy encourages use of a ‘treatment buddy’ to support adherence; however, this is not considered a requirement for initiation onto treatment (South African Department of Health 2010). Nearly all HIV-only participants reported that a guardian accompanied them to their most recent PHC clinic visit. Participants with TB-only were not as frequently accompanied to the PHC clinic and therefore had relatively lower costs.

Our study supports previous findings that the primary drivers of TB patient costs are income and job loss associated with time spent care-seeking and inability to work due to illness (Muniyandi *et al.* 2005; Aspler *et al.* 2008; Ukwaja *et al.* 2013; Chimbindi *et al.* 2015). All people with TB had high numbers of health facility visits and these were reflected in time and travel costs. Study participants with TB also had a high rate of job loss, no matter their HIV status. South Africa is currently scaling-up community based approaches to treatment supervision that may reduce these costs in the future.

Our study also supports previous findings that supplementary food is an important driver of TB patient costs in South Africa (Bond *et al.* 2008; Foster *et al.* 2015), raising the question of whether patients are getting appropriate education regarding nutrition and TB. Previous studies have indicated that patients may perceive that TB and HIV drugs must be supplemented with higher food intake, often including foods outside of the normal South African diet including eggs, fruit, soft drinks, and meat (Bond *et al.* 2008). Improved nutrition counselling for people with TB and/or HIV is needed to help households meet dietary needs within their normal spending capabilities.

As with any patient-level costing effort, this study faced several methodological limitations. Primarily, our comparisons are made on a small sample and the participant groups we compared did not have equal numbers of participants because eligible participants were recruited consecutively, and the MERGE study had fewer participants with TB. Methodological choices taken in this study, and the potential limitations of these are discussed in detail by Sweeney et al. (2016). In practice, when conducting patient cost interviews alongside intervention studies and trials, analysts are faced with either obtaining comprehensive costs of a smaller sample or limited costs (usually OOP) from a larger sample. Due to the importance of indirect costs as highlighted by previous studies, we chose the former. We chose a recall period of 3–5 months; this poses some risk of recall bias, which we weighed against the potential to miss costs. Second, the patient costs questionnaire was time consuming taking up to 60 min. The long survey times required pose some risk of survey fatigue for interviewees, as well as interviewers. A number of training sessions were conducted with the interviewers and a number of recruitment guides were developed to make the recruitment process more feasible. Finally there is considerable debate in the literature surrounding the measurement of indirect costs, and the approach taken in previous studies is inconsistent (Zhang *et al.* 2011; Krol *et al.* 2013; Krol and Brouwer 2014; Laurence *et al.* 2015). We chose to report income loss as our primary measure of indirect cost in order to avoid double-counting and possible bias against people with zero income, and report on time loss separately to facilitate comparison with other studies (Wingfield *et al.* 2014; Chimbindi *et al.* 2015). Further methodological research on measurement of indirect costs would facilitate future analyses of patient costs.

## Conclusions

Given the catastrophic costs associated with TB and HIV, even in settings where TB and HIV treatment are provided for ‘free’, social and income protection policies are likely to be required to protect these patients if global targets on catastrophic cost reduction are to be met. Integration of services has potential to reduce the number of visits to the health facility, and our data shows patients are receiving this care already in South Africa. However, we also find that those with TB/HIV suffer the highest costs, and integration should be further extended to ensure that both the economic burden of ill-health and that of treatment are minimized for vulnerable households.

## Supplementary Material

Supplementary Appendix Figure I

Supplementary Appendix Table I

Supplementary Appendix Table II
