# Supplementary material for: The patient costs of care for those with TB and HIV: a cross-sectional study from South Africa
Source: Health Policy Plan. 2017 Feb 15;32(Suppl 4):iv48–56. doi: 10.1093/heapol/czw183 (PMC5886108; doi:10.1093/heapol/czw183)
Supplement: Supplementary Appendix Figure I [file czw183_appendix_figure_i_catastrophic_costs_due_to_illness.docx]

Appendix Figure I Catastrophic costs due to illness, by patient group (direct costs only)
