# Supplementary material for: The patient costs of care for those with TB and HIV: a cross-sectional study from South Africa
Source: Health Policy Plan. 2017 Feb 15;32(Suppl 4):iv48–56. doi: 10.1093/heapol/czw183 (PMC5886108; doi:10.1093/heapol/czw183)
Supplement: Supplementary Appendix Table I [file czw183_appendix_table_i_detailed_health_service_use_at_all_facility_types.docx]

**Appendix Table I Detailed health service use at all facility types, by participant group**

|  | Number of participants | | | Mean number of visits in post diagnosis period | | |
| --- | --- | --- | --- | --- | --- | --- |
| Participant group | **TB/HIV**  **(n = 116)** | **TB only**  **(n = 40)** | **HIV only**  **(n = 298)** | **TB/HIV**  **(n = 116)** | **TB only**  **(n = 40)** | **HIV only**  **(n = 298)** |
| Study Clinic |  |  |  |  |  |  |
| TB/HIV visits | 91 | 27 | 0 | 6.4 | 1.1 |  |
| TB visits | 95 | 39 | 0 | 13.3 | 14.9 |  |
| HIV visits | 52 | 2 | 298 | 4.4 | 1.0 | 4.6 |
| Other Clinic |  |  |  |  |  |  |
| TB visits | 6 | 5 | 0 | 1.3 | 4.4 |  |
| HIV visits | 2 | 2 | 7 | 1.5 | 1.0 | 1.6 |
| Pharmacy |  |  |  |  |  |  |
| TB visits | 2 | 0 | 0 | 1.5 |  |  |
| HIV visits | 2 | 0 | 10 | 1.5 |  | 1.2 |
| General Practitioner |  |  |  |  |  |  |
| TB visits | 4 | 1 | 0 | 1.8 | 1.0 |  |
| HIV visits | 3 | 0 | 20 | 3.3 |  | 1.6 |
| Hospital (inpatient) |  |  |  |  |  |  |
| TB visits | 10 | 0 | 0 | 1.2 |  |  |
| HIV visits | 3 | 0 | 2 | 1.0 |  | 1.0 |
| Hospital (outpatient) |  |  |  |  |  |  |
| TB visits | 3 | 0 | 0 | 2.0 |  |  |
| HIV visits | 5 | 0 | 0 | 1.2 |  |  |
| Traditional Healer |  |  |  |  |  |  |
| TB visits | 1 | 0 | 0 | 1.0 |  |  |
| HIV visits | 0 | 0 | 9 |  |  | 1.7 |
