# Supplementary material for: The patient costs of care for those with TB and HIV: a cross-sectional study from South Africa
Source: Health Policy Plan. 2017 Feb 15;32(Suppl 4):iv48–56. doi: 10.1093/heapol/czw183 (PMC5886108; doi:10.1093/heapol/czw183)
Supplement: Supplementary Appendix Table II [file czw183_appendix_table_ii_detailed_costs_at_all_facility_types.docx]

**Appendix Table II – Detailed costs for all facility types, by participant group**

|  |  | | | TB/HIV  (n=116) | | TB only  (n=40) | | HIV only  (n=298) | |
| --- | --- | --- | --- | --- | --- | --- | --- | --- | --- |
|  |  | | | **mean** | **(SD)** | **Mean** | **(SD)** | **mean** | **(SD)** |
| Direct costs | Medical | Study clinic | | 0.00 | 0.00 | 0.00 | 0.00 | 0.00 | 0.00 |
|  |  | Other clinic | | 0.00 | 0.00 | 0.00 | 0.00 | 0.00 | 0.00 |
|  |  | Pharmacy | | 0.00 | 0.01 | 0.07 | 0.42 | 0.00 | 0.00 |
|  |  | General practitioner | | 0.30 | 2.72 | 0.00 | 0.00 | 0.82 | 4.13 |
|  |  | Hospital-outpatient | | 0.11 | 0.70 | 0.00 | 0.00 | 0.00 | 0.00 |
|  |  | Hospital-inpatient | | 0.32 | 2.28 | 0.00 | 0.00 | 0.01 | 0.14 |
|  |  | Traditional healer | | 1.00 | 9.67 | 0.00 | 0.00 | 0.05 | 0.44 |
|  | Travel | Patient | Study clinic | 4.12 | 8.91 | 1.69 | 3.31 | 1.25 | 3.07 |
|  |  |  | Other clinic | 0.02 | 0.12 | 0.05 | 0.20 | 0.02 | 0.15 |
|  |  |  | Pharmacy | 0.00 | 0.00 | 0.00 | 0.00 | 0.04 | 0.35 |
|  |  |  | General practitioner | 0.30 | 2.70 | 0.00 | 0.00 | 0.13 | 1.10 |
|  |  |  | Hospital-outpatient | 0.21 | 0.93 | 0.00 | 0.00 | 0.00 | 0.00 |
|  |  |  | Hospital-inpatient | 0.06 | 0.43 | 0.00 | 0.00 | 0.03 | 0.60 |
|  |  |  | Traditional healer | 0.04 | 0.48 | 0.00 | 0.00 | 0.01 | 0.13 |
|  |  | Guardian | Study clinic | 0.43 | 2.37 | 0.00 | 0.00 | 0.27 | 2.78 |
|  |  |  | Other clinic | 0.00 | 0.00 | 0.00 | 0.00 | 0.01 | 0.08 |
|  |  |  | Pharmacy | 0.00 | 0.00 | 0.00 | 0.00 | 0.01 | 0.18 |
|  |  |  | General practitioner | 0.25 | 2.65 | 0.00 | 0.00 | 0.05 | 0.81 |
|  |  |  | Hospital-outpatient | 0.12 | 0.83 | 0.00 | 0.00 | 0.00 | 0.00 |
|  |  |  | Hospital-inpatient | 0.14 | 1.45 | 0.00 | 0.00 | 0.10 | 1.28 |
|  |  |  | Traditional healer | 0.00 | 0.00 | 0.00 | 0.00 | 0.01 | 0.09 |
|  | Food | Hospital | | 0.26 | 1.31 | 0.00 | 0.00 | 0.04 | 0.47 |
|  |  | Special foods | | 13.14 | 17.33 | 8.06 | 11.05 | 9.76 | 14.91 |
|  | Loan interest | | | 0.93 | 9.78 | 0.00 | 0.00 | 5.68 | 89.11 |
|  | **Total direct** | | | **21.72** | **29%^1^** | **9.86** | **14%** | **18.28** | **45%^1^** |
| Indirect Costs | Patient Income Loss | Job loss income loss | | 15.40 | 126.17 | 17.78 | 76.69 | 2.99 | 24.30 |
|  |  | Care-seeking income loss | | 30.45 | 105.56 | 34.60 | 98.99 | 13.81 | 59.03 |
|  | Opportunity Costs of Time | Guardian | Study clinic | 1.13 | 4.58 | 0.23 | 1.00 | 3.92 | 3.35 |
|  |  |  | Other clinic | 0.00 | 0.00 | 0.03 | 0.17 | 0.03 | 0.36 |
|  |  |  | Pharmacy | 0.00 | 0.00 | 0.01 | 0.06 | 0.00 | 0.04 |
|  |  |  | General practitioner | 0.03 | 0.32 | 0.00 | 0.00 | 0.04 | 0.41 |
|  |  |  | Hospital-outpatient | 0.05 | 0.65 | 0.00 | 0.00 | 0.00 | 0.00 |
|  |  |  | Hospital-inpatient | 0.88 | 6.19 | 0.00 | 0.00 | 0.13 | 1.11 |
|  |  |  | Traditional healer | 0.00 | 0.00 | 0.00 | 0.00 | 0.01 | 0.16 |
|  |  | Carer | | 4.42 | 11.35 | 5.81 | 13.52 | 1.19 | 5.77 |
|  | **Total indirect** | | | **52.34** | **71%^1^** | **58.47** | **86%** | **22.13** | **55%^1^** |
| Grand total | | | | **74.07** |  | **68.33** |  | **40.41** |  |

^1^ percentage of the overall total
